# Associations of Diabetes, Smoking, and Metabolic Factors With the Risk of Breast and Prostate Cancers: A Population‐Based Retrospective Cohort Study

**DOI:** 10.1002/cam4.71556

**Published:** 2026-01-26

**Authors:** Sarah Tsz Yui Yau, Chi Tim Hung, Eman Yee Man Leung, Albert Lee, Eng Kiong Yeoh

**Affiliations:** ^1^ JC School of Public Health and Primary Care The Chinese University of Hong Kong Hong Kong China

**Keywords:** breast cancer, diabetes, metabolism, prostate cancer, smoking

## Abstract

**Background:**

Previous studies reported an inverse association between diabetes and prostate cancer. Moreover, the associations of smoking with breast and prostate cancers remain inconclusive. This study aims to investigate whether diabetes, smoking, or metabolic factors are associated with female breast and prostate cancers incidence, and explore factors associated with their prevalence.

**Methods:**

In this retrospective cohort study, patients who utilized public healthcare services between year 2000 and 2021 in Hong Kong were identified. For cancer incidence, patients (*n* = 192,456) were followed up until a diagnosis of cancer. Factors associated with site‐specific cancer incidence were assessed using Cox regression, whereas factors associated with site‐specific cancer prevalence were examined using logistic regression.

**Results:**

Patients with diabetes did not have a higher risk of developing breast (adjusted hazard ratio [aHR]: 1.11, 95% CI = 0.86–1.42) or prostate cancer (aHR: 0.94, 95% CI = 0.72–1.21) when compared to those without diabetes. The associations with breast cancer remained consistent when stratified by menopausal status. Smoking was associated with an increased risk of prostate cancer (aHR: 1.16, 95% CI = 1.04–1.30) but not for breast cancer. However, male patients who had diabetes, elevated lipid levels, haematuria, or cystitis were more likely to have a previous prostate cancer diagnosis.

**Conclusion:**

Findings indicate no significant association between diabetes and incidence of breast or prostate cancer, but suggest smoking as a potential risk factor for prostate cancer.

## 1 Introduction

Globally, female breast and prostate cancers rank second and fourth in cancer incidence respectively, contributing to almost 3,800,000 new cases in year 2022 [1]. Past research has shown that diabetes is associated with a number of site‐specific cancers potentially due to underlying biological factors under diabetes condition or overlapping risk factors between diabetes and cancer [2, 3].

Previous studies mostly found that diabetes is associated with an increased risk of female breast cancer but a decreased risk of prostate cancer [2, 3]. In epidemiological studies, the positive association between diabetes and breast cancer was shown in postmenopausal females, but not in premenopausal females [4, 5]. Similarly, the inverse association between diabetes and prostate cancer was demonstrated for nonaggressive prostate cancer, but not for aggressive type [6, 7], where aggressiveness is normally defined as advanced stage or high grade using TNM staging and Gleason score respectively [8]. Nevertheless, female breast cancer and diabetes are both associated with adiposity [9] and their temporal association is equivocal [3]. In addition, low testosterone in diabetes is proposed as a mechanism to explain the observed inverse association between diabetes and prostate cancer [2, 3, 10, 11]. Nevertheless, previous studies yielded conflicting results on the association between testosterone and prostate cancer [12]. Furthermore, prostate tumor cells appear to exhibit differential responses to androgens over the course of carcinogenesis [13, 14].

Moreover, while smoking is known as a carcinogenic agent to many organs in humans, evidence for smoking as a risk factor for breast cancer remains limited, and there is a lack of evidence for its association with prostate cancer [15]. Furthermore, smoking may increase circulating androgen levels in both females [16, 17, 18] and males [19], potentially influencing the risk of hormone‐related cancers. However, the prevalence of smoking is generally low in the female population, rendering lower statistical power in analyses. In addition, given that diabetes is associated with an elevated risk of cancer [2, 3], it is possible that hyperglycemia [2, 3], insulin‐and‐insulin like growth factor (IGF) axis [2, 3], or metabolic dysfunction may play a role in influencing the risk of breast or prostate cancer. Nevertheless, it has been shown that unlike most solid tumors, the Warburg effect is not observed in prostate cancer cells [20]. Furthermore, given the role of hormone receptor expression in breast and prostate cancer and the possible role of diabetes and metabolic dysfunction in cancer development, the associations between lipid profile and cancers of the female breast or prostate remain inconclusive [21, 22, 23, 24].

Generally, diabetes and smoking demonstrate positive individual associations with many site‐specific cancers. Their apparent inverse associations with prostate cancer in some studies are unexpected and biologically perplexing. Furthermore, given the hormonal changes over the reproductive years, earlier age of onset in female breast cancer, and differential responses towards hormone therapy over the course of disease progression in prostate cancer [13, 14], the associations of diabetes, metabolic factors, and smoking with female breast and prostate cancers may exhibit different patterns from other site‐specific cancers.

This study seeks to (i) examine whether diabetes, smoking, or metabolic factors are associated with the subsequent risk of female breast and prostate cancer incidence; and (ii) explore factors associated with their prevalence, controlling for available information from electronic health records in an Asian population.

## 2 Methods

### 2.1 Study Design and Study Population

This retrospective cohort study was performed using territory‐wide electronic health records of Hong Kong. The Hospital Authority (HA) is a statutory body responsible for providing public healthcare services. The HA maintains a centralized clinical data repository on patient demographics, disease diagnoses, prescription records, laboratory measurements, clinical notes, and radiology reports. Data used in this study were linked to death records from the Immigration Department. Disease diagnoses were coded according to the International Classification of Disease 10th revision (ICD‐10), or the International Classification of Primary Care 2nd edition (ICPC‐2). Individual‐level data across datasets were linked via pseudonymous identifiers. Data were accessed via HA Data Collaboration Lab.

The Hong Kong government did not launch population‐based screening programmes for female breast or prostate cancer during the study period (up to year 2021). However, the government is currently conducting a phase II breast cancer screening pilot programme beginning in June 2025 to subsidize high‐risk females to undergo screening.

### 2.2 Patients

Patients who utilized public healthcare services between year 2000 and 2021 with at least one record on selected laboratory measurements (fasting glucose, low‐density lipoprotein [LDL] cholesterol, high‐density lipoprotein [HDL] cholesterol, triglycerides, and alanine aminotransferase [ALT]) were initially included. Prescription records of anti‐diabetic drugs were extracted to identify diabetes status of patients. Patients with and without diabetes were followed up since diabetes onset (using initiation of anti‐diabetic drugs as a proxy) and the earliest record of selected laboratory measurements respectively. Those with less than two records of any of the selected laboratory measurements within one year of the index date were excluded. A total of 161,790 patients with diabetes and 67,777 patients without diabetes were then included. To exclude possible type 1 diabetes cases, patients who (i) received a diagnosis of diabetes below 30 years old [25, 26]; or (ii) received a diagnosis of diabetes below 60 years old [25] and insulin treatment only within one year of diabetes onset (but without taking any other anti‐diabetic drugs during study period) were excluded (*n* = 3018). Similarly, for patients without diabetes, those below 30 years old at baseline were also excluded (*n* = 2205). Of the 11,486 patients who had a history of cancer at baseline, 2244 female patients who had a prior diagnosis of breast cancer and 1020 males who had a prior diagnosis of prostate cancer within two years before the index date were identified. Patients below 30 years old with a history of cancer at baseline were excluded from further analyses (*n* = 19).

**FIGURE 1:**
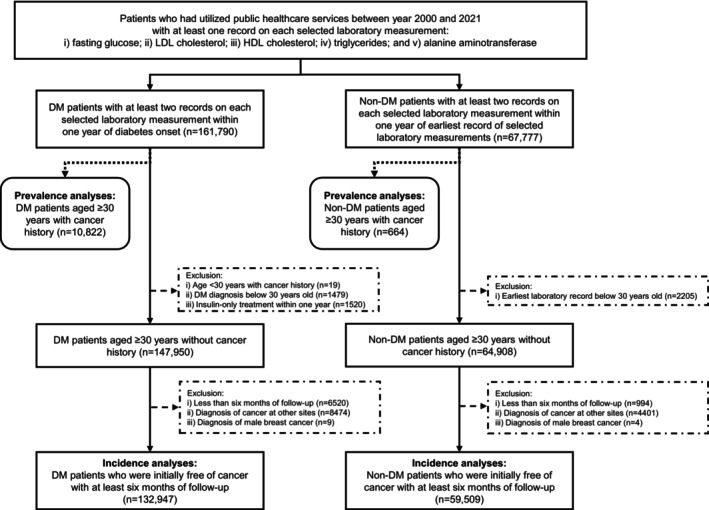
Flow chart of patient selection procedures. DM, diabetes mellitus; HDL, high‐density lipoprotein; LDL, low‐density lipoprotein.

To examine the association between diabetes and cancer incidence, patients with a history of cancer at baseline were excluded in subsequent analyses. A total of 147,950 patients with diabetes and 64,908 patients without diabetes (predominantly type 2) who were initially free of cancer were then included. Patients with and without diabetes were followed up until a cancer diagnosis, death, or December 31st, 2021, whichever occurred earlier. For both patients with and without diabetes, to minimize reverse causality, those who had less than six months of follow‐up [3] were excluded (*n* = 7514). In addition, those who were diagnosed with cancer at sites other than breast (female only) or prostate during follow‐up (*n* = 12,888) were excluded. Male breast cancer cases (*n* = 13) were excluded. In other words, only females who subsequently developed breast cancer, males who subsequently developed prostate cancer, and those who remained cancer‐free during follow‐up were included. Finally, a total of 132,947 patients with diabetes and 59,509 patients without diabetes were included. Figure 1 shows the flow chart of patient selection procedures.

### 2.3 Outcomes

The outcomes of interest were diagnosis of female breast cancer (ICD‐10: C50, excluding male) and prostate cancer (ICD‐10: C61).

### 2.4 Independent Variables

Independent variables included diabetes, metabolic factors, behavior (smoking), demographics (age at baseline and sex), medical history, and medication use. Diabetes was defined as use of anti‐diabetic drugs (metformin, sulfonylurea, insulin, dipeptidyl peptidase‐4 inhibitors, acarbose, meglitinide, glitazone, sodium‐glucose cotransporter‐2 inhibitors, and glucagon‐like peptide‐1 receptor agonists). Metabolic factors included fasting glucose, lipids (LDL cholesterol, HDL cholesterol, and triglycerides), and liver enzyme (ALT). Laboratory measurements were taken as the mean of at least two records nearest to the baseline within one year. Smoking status was extracted from clinical notes since smoking status is frequently recorded in clinical notes by clinicians during their encounters with patients. To minimize misclassification bias, smoking status was collapsed into two levels, namely ever and never smoking. Medical history included urinary symptoms (haematuria and cystitis) and common medical conditions such as chronic obstructive pulmonary disease, pneumonia, tuberculosis, ischemic heart disease, cerebrovascular disease, heart failure, chronic viral hepatitis, liver cirrhosis, and fatty liver. Disease diagnoses were determined from inpatient and outpatient diagnosis codes (Table S1). The presence of fatty liver was determined from diagnosis codes and radiology reports (ultrasonography, computed tomography, and magnetic resonance imaging). Medication included anti‐diabetic drugs, aspirin, nonsteroidal anti‐inflammatory drugs, anti‐coagulants, anti‐platelets, statins, anti‐hypertensive drugs (alpha‐blockers, angiotensin‐converting enzyme inhibitors, angiotensin receptor blockers, beta‐blockers, calcium channel blockers, and diuretics). Medication use was defined as whether patients had taken a drug at baseline.

### 2.5 Data Analysis

Baseline characteristics of patients by subsequent female breast and prostate cancer status were presented in mean with standard deviation or median with interquartile range for continuous variables, and count with proportion for categorical variables. Factors associated with site‐specific cancer incidence were examined using Cox proportional hazards regression and reported in adjusted hazard ratio (aHR) with 95% confidence interval (CI). The studied factors included diabetes, smoking, and metabolic factors (serum glucose, lipids, and ALT). For the female population, stratified analyses by menopausal status were performed using age of 50 years as a proxy for menopause [27, 28, 29, 30]. Separately, factors associated with site‐specific cancer prevalence (cancer diagnosis within two years before the index date) were examined using logistic regression [26] and reported in adjusted odds ratio (aOR) with 95% CI. Given the possible higher prevalence of prostate cancer among males after a diagnosis [31] or evaluation [32] for haematuria, the associations between urinary symptoms (haematuria and cystitis) and site‐specific cancer prevalence were also explored.

## 3 Results

Of the 192,456 patients without cancer history, 1493 and 1267 developed incident female breast and prostate cancers during follow‐up (median: 6.33 years) respectively. The corresponding overall incidence rates were 2.39 and 1.72 per 1000 person‐years. Among the 132,947 patients with diabetes, 945 and 735 patients developed incident female breast and prostate cancers respectively (incidence rates: 2.16 and 1.46 per 1000 person‐years). On the other hand, among the 59,509 patients without diabetes, 548 and 532 patients developed incident female breast and prostate cancers respectively (incidence rates: 2.95 and 2.25 per 1000 person‐years). Table 1 shows the baseline characteristics of patients who developed incident female breast or prostate cancer, and those who remained free of cancer during follow‐up. The proportions of females who had ever smoked were the same irrespective of their subsequent breast cancer status (4.09%). However, males who subsequently developed prostate cancer were more likely to be an ever smoker than those who remained free of cancer during follow‐up (39.15 vs. 30.90%, *p* < 0.001).

**TABLE 1:** Baseline characteristics of patients by subsequent female breast and prostate cancer status.

Characteristics	Female		Male	
	Breast	No cancer	Prostate	No cancer
	(*n* = 1493)	(*n* = 86,483)	(*n* = 1267)	(*n* = 103,213)
Demographics				
Age at baseline in year, mean ± SD	60.96 ± 10.48	62.14 ± 12.61	67.13 ± 8.55	60.82 ± 11.94
Follow‐up time in month, median (IQR)	56 (26–95)	77 (39–124)	57 (26–100.5)	76 (39–122)
Metabolic factors				
Diabetes, *n* (%)	945 (63.30%)	60,850 (70.36%)	735 (58.01%)	70,417 (68.22%)
Fasting glucose in mmol/L, mean ± SD	7.03 ± 2.19	7.24 ± 2.33	6.89 ± 2.18	7.31 ± 2.40
Low‐density lipoprotein cholesterol in mmol/L, mean ± SD	3.12 ± 0.89	2.99 ± 0.90	2.93 ± 0.87	2.84 ± 0.87
High‐density lipoprotein cholesterol in mmol/L, mean ± SD	1.38 ± 0.36	1.38 ± 0.35	1.23 ± 0.33	1.19 ± 0.31
Triglycerides in mmol/L, mean ± SD	1.67 ± 1.13	1.64 ± 1.01	1.59 ± 1.41	1.70 ± 1.29
Alanine transaminase in U/L, mean ± SD	32.23 ± 57.32	32.02 ± 47.22	32.48 ± 38.70	37.57 ± 68.32
Behavior				
Ever smoker, *n* (%)	61 (4.09%)	3533 (4.09%)	496 (39.15%)	31,895 (30.90%)
Medical history				
Haematuria, *n* (%)	34 (2.28%)	1915 (2.21%)	41 (3.24%)	2940 (2.85%)
Cystitis, *n* (%)	74 (4.96%)	4803 (5.55%)	20 (1.58%)	1390 (1.35%)
Chronic obstructive pulmonary disease, *n* (%)	0 (0%)	131 (0.15%)	6 (0.47%)	1015 (0.98%)
Pneumonia, *n* (%)	1 (0.07%)	97 (0.11%)	2 (0.16%)	252 (0.24%)
Tuberculosis, *n* (%)	2 (0.13%)	148 (0.17%)	4 (0.32%)	528 (0.51%)
Ischemic heart disease, *n* (%)	12 (0.80%)	1173 (1.36%)	42 (3.31%)	3592 (3.48%)
Cerebrovascular disease, *n* (%)	45 (3.01%)	2657 (3.07%)	47 (3.71%)	3905 (3.78%)
Heart failure, *n* (%)	30 (2.01%)	2756 (3.19%)	37 (2.92%)	3345 (3.24%)
Chronic viral hepatitis B, *n* (%)	9 (0.60%)	776 (0.90%)	13 (1.03%)	1284 (1.24%)
Chronic viral hepatitis C, *n* (%)	2 (0.13%)	85 (0.10%)	0 (0%)	171 (0.17%)
Liver cirrhosis, *n* (%)	2 (0.13%)	109 (0.13%)	3 (0.24%)	330 (0.32%)
Fatty liver, *n* (%)	26 (1.74%)	1553 (1.80%)	6 (0.47%)	1593 (1.54%)
Medication use				
Anti‐diabetic drugs				
Metformin, *n* (%)	775 (51.91%)	51,210 (59.21%)	554 (43.73%)	56,784 (55.02%)
Sulfonylurea, *n* (%)	241 (16.14%)	12,907 (14.92%)	247 (19.49%)	19,478 (18.87%)
Insulin, *n* (%)	72 (4.82%)	7093 (8.20%)	67 (5.29%)	10,926 (10.59%)
Dipeptidyl peptidase‐4 inhibitors, *n* (%)	5 (0.33%)	623 (0.72%)	7 (0.55%)	999 (0.97%)
Acarbose, *n* (%)	1 (0.07%)	106 (0.12%)	0 (0%)	123 (0.12%)
Meglitinide, *n* (%)	0 (0%)	2 (0.00%)	0 (0%)	0 (0%)
Glitazone, *n* (%)	3 (0.20%)	251 (0.29%)	2 (0.16%)	390 (0.38%)
Sodium‐glucose cotransporter‐2 inhibitors, *n* (%)	0 (0%)	220 (0.25%)	2 (0.16%)	487 (0.47%)
Glucagon‐like peptide‐1 receptor agonists, *n* (%)	0 (0%)	30 (0.03%)	0 (0%)	27 (0.03%)
Aspirin, *n* (%)	330 (22.10%)	22,208 (25.68%)	457 (36.07%)	36,915 (35.77%)
Nonsteroidal anti‐inflammatory drugs, *n* (%)	782 (52.38%)	51,299 (59.32%)	532 (41.99%)	51,375 (49.78%)
Anti‐coagulants, *n* (%)	72 (4.82%)	6314 (7.30%)	103 (8.13%)	11,918 (11.55%)
Anti‐platelets, *n* (%)	334 (22.37%)	22,546 (26.07%)	471 (37.17%)	37,495 (36.33%)
Statins, *n* (%)	538 (36.03%)	37,190 (43.00%)	518 (40.88%)	47,259 (45.79%)
Alpha‐blockers, *n* (%)	46 (3.08%)	2879 (3.33%)	343 (27.07%)	16,246 (15.74%)
Angiotensin‐converting enzyme inhibitors, *n* (%)	419 (28.06%)	25,549 (29.54%)	400 (31.57%)	34,790 (33.71%)
Angiotensin receptor blockers, *n* (%)	100 (6.70%)	8636 (9.99%)	84 (6.63%)	8768 (8.50%)
Beta‐blockers, *n* (%)	592 (39.65%)	32,964 (38.12%)	452 (35.67%)	37,524 (36.36%)
Calcium channel blockers, *n* (%)	671 (44.94%)	42,944 (49.66%)	535 (42.23%)	48,373 (46.87%)
Diuretics, *n* (%)	279 (18.69%)	17,450 (20.18%)	210 (16.57%)	16,961 (16.43%)

Abbreviations: IQR, interquartile range; SD, standard deviation.

Among the 10,822 patients with diabetes who were 30 years old or above at diabetes diagnosis and had a history of cancer, 2110 and 991 patients were diagnosed with female breast and prostate cancers within two years before diabetes diagnosis, respectively. Among the 664 patients without diabetes who were 30 years old or above at index date and had a history of cancer, 134 and 29 patients had a history of female breast and prostate cancers within two years before the index date, respectively.

### 3.1 Diabetes, Smoking, Metabolic Factors, and Cancer Incidence

**FIGURE 2:**
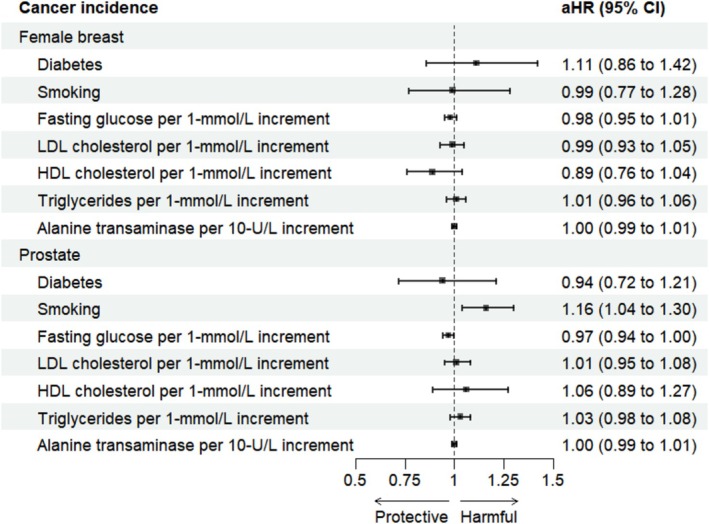
Forest plot of the hazard ratios of diabetes, smoking, and metabolic factors on female breast and prostate cancers. AHR, adjusted hazard ratio; CI, confidence interval; HDL, high‐density lipoprotein; HR, hazard ratio; LDL, low‐density lipoprotein. All models were adjusted for age, smoking, metabolic factors, medical history (chronic obstructive pulmonary disease, pneumonia, tuberculosis, ischemic heart disease, cerebrovascular disease, heart failure, chronic viral hepatitis, liver cirrhosis, fatty liver, hematuria, and cystitis) and medication use (anti‐diabetic drugs, aspirin, nonsteroidal anti‐inflammatory drugs, anti‐coagulants, anti‐platelets, statins, and anti‐hypertensive drugs).

Among the female population, patients with diabetes were of no statistical difference in developing breast cancer when compared to those without diabetes (aHR 1.11, 95% CI: 0.86–1.42). When stratified by menopausal status (using age of 50 years as a proxy), the null associations between diabetes and breast cancer remained unchanged. Neither smoking nor metabolic factors (glucose and lipids) were associated with the risk of breast cancer incidence (Figure 2).

Among the male population, patients with diabetes did not have a statistically different risk of developing prostate cancer when compared to those without diabetes (aHR 0.94, 95% CI: 0.72–1.21). Smoking was linked to an elevated risk of prostate cancer (aHR 1.16, 95% CI: 1.04–1.30 ever vs. never smoker). However, metabolic factors were also not associated with the risk of prostate cancer incidence (Figure 2).

No association between urinary symptoms (haematuria or cystitis) and incidence of breast or prostate cancer was found.

### 3.2 Diabetes, Lipid Profile, Urinary Symptoms, and Cancer Prevalence

Female patients who had diabetes (aOR 5.60, 95% CI: 4.40–7.13), lower LDL cholesterol (aOR 0.82, 95% CI: 0.77–0.87 per 1‐mmol/L increment), or higher triglycerides (aOR 1.06, 95% CI: 1.02–1.09 per 1‐mmol/L increment) were more likely to have a previous breast cancer diagnosis. On the other hand, male patients who had diabetes (aOR 6.38, 95% CI: 4.15–9.81), higher levels of LDL cholesterol (aOR 1.15, 95% CI: 1.06–1.24 per 1‐mmol/L increment), HDL cholesterol (aOR 1.79, 95% CI: 1.49–2.16 per 1‐mmol/L increment), or triglycerides (aOR 1.08, 95% CI: 1.04–1.11 per 1‐mmol/L increment), haematuria (aOR 2.07, 95% CI: 1.74–2.47), or cystitis (aOR 1.99, 95% CI: 1.59–2.47) were more likely to have a previous prostate cancer diagnosis (Table 2).

**TABLE 2:** Factors associated with site‐specific cancer prevalence (diagnosis before study entry).

Factor	Female breast	Prostate
	aOR (95% CI)	aOR (95% CI)
Demographical and behavioral factors		
Age at baseline, per 10‐year increment	**1.12 (1.07–1.16)**	**2.26 (2.10–2.43)**
Ever smoker	0.82 (0.65–1.02)	1.01 (0.88–1.15)
Metabolic factors		
Diabetes	**5.60 (4.40–7.13)**	**6.38 (4.15–9.81)**
Fasting glucose, per 1‐mmol/L increment	0.99 (0.97–1.02)	1.01 (0.98–1.05)
Low‐density lipoprotein cholesterol, per 1‐mmol/L increment	**0.82 (0.77–0.87)**	**1.15 (1.06–1.24)**
High‐density lipoprotein cholesterol, per 1‐mmol/L increment	0.98 (0.86–1.12)	**1.79 (1.49–2.16)**
Triglycerides, per 1‐mmol/L increment	**1.06 (1.02–1.09)**	**1.08 (1.04–1.11)**
Alanine transaminase, per 10‐U/L increment	1.00 (1.00–1.01)	1.00 (0.99–1.01)
Urinary symptoms		
Haematuria	0.98 (0.77–1.24)	**2.07 (1.74–2.47)**
Cystitis	1.00 (0.85–1.17)	**1.99 (1.59–2.47)**

*Note:* All models were adjusted for age, smoking, metabolic factors, medical history (chronic obstructive pulmonary disease, pneumonia, tuberculosis, ischemic heart disease, cerebrovascular disease, heart failure, chronic viral hepatitis, liver cirrhosis, fatty liver, hematuria and cystitis) and medication use (anti‐diabetic drugs, aspirin, nonsteroidal anti‐inflammatory drugs, anti‐coagulants, anti‐platelets, statins and anti‐hypertensive drugs).

Abbreviations: aOR, adjusted odds ratio; CI, confidence interval. aOR in bold indicates statistical significance at an alpha level of 0.05.

## 4 Discussion

Using electronic health records of an Asian population, the current study did not find statistically significant associations between diabetes and incidence of female breast or prostate cancer, controlling for age, smoking, metabolic factors, disease diagnoses, and medication use. Nevertheless, findings support smoking as a potential risk factor for prostate cancer. Also, patients who had diabetes or altered lipid profile were more likely to have a diagnosis of female breast or prostate cancer. In particular, male patients with elevated lipids or urinary symptoms (haematuria or cystitis) were more likely to have a diagnosis of prostate cancer.

Prior research largely supports the association between diabetes and an increased risk of female breast cancer [2, 4, 5], in particular after menopause [4, 5]. Nevertheless, some studies did not find a significant association between diabetes and breast cancer, regardless of menopausal status [29, 33, 34]. Several postulated mechanisms linking diabetes to breast cancer include activation of insulin and IGF pathways and dysregulation of endogenous sex hormones [35]. In the present study, diabetes was not found to be associated with the risk of female breast cancer incidence after accounting for age, metabolic profile, smoking status, medical history and medication use. The results remained consistent when stratified by menopausal status using age of 50 years as a proxy for menopause. The differences in findings could be potentially attributed to differences in study design and confounding control. This study lacks information on adiposity indicators [5]. Nevertheless, prior research [5] did not find an overall statistical difference in risk estimates adjusted for body mass index (BMI) and those that were not. Moreover, adjustment for BMI tended to attenuate the risk towards a null association [5].

On the other hand, previous research mostly found an inverse association between diabetes and prostate cancer [2, 10, 11, 36, 37, 38, 39], in particular for nonaggressive type [6, 7]. Some proposed mechanisms for the association include disturbances in insulin and IGF pathways [10, 11] as well as alterations in sex hormones [6, 7, 10]. Previous studies found that lower testosterone levels are associated with obesity [40] and type 2 diabetes in males [36, 41, 42]. A recent study further suggests an inverse association between testosterone levels and mortality risk in males, particularly at older ages [43]. Conversely, higher testosterone levels are associated with obesity [44] and type 2 diabetes in females [42]. Low testosterone in diabetes may potentially account for the observed inverse associations between diabetes and prostate cancer in previous studies [10, 11, 37]. However, although it is thought that testosterone may promote carcinogenesis of the prostate, studies investigating the association between testosterone and prostate cancer produced conflicting results [12], and androgen deprivation may only manage prostate cancer growth at early stages [14], implying that lower testosterone in diabetes may not fully explain the inverse association. The current study did not find an overall significant association between diabetes and prostate cancer incidence.

While smoking is a known risk factor for many cancers [15], its associations with breast cancer and prostate cancer remain controversial. According to the International Agency for Research on Cancer by the World Health Organization, limited evidence is available to support tobacco smoking as a carcinogenic agent to the breast in humans [15]. Earlier review concluded no association between smoking and breast cancer [45]. The association between smoking and breast cancer remains less consistent [46] or stratified by menopausal status [47]. Some evidence supports the positive association between smoking and breast cancer risk in premenopausal females [47]. Findings of the present study did not suggest an association between smoking and female breast cancer, even when stratified by menopausal status. On the other hand, the association between smoking and prostate cancer appears to be inverse in most observational studies [48, 49, 50, 51]. Nevertheless, some studies found no overall association [52], or a positive association when stratified by smoking dosage [52, 53] or cancer aggressiveness [49, 51, 52, 54]. Specifically, a number of studies have found that current smoking is associated with an elevated risk of mortality from prostate cancer [49, 51, 52, 54]. This is consistent with the finding of smoking as a risk factor for prostate cancer incidence in the present study. Previous research suggests that smoking may elevate circulating androgen levels in premenopausal [16, 17] and postmenopausal [18] females, and males [19]. However, it was reported that smoking, obesity, and diabetes have an individual and joint negative association with prostate‐specific antigen (PSA) level [55]. This may potentially influence referral patterns for further prostate cancer testing.

Moreover, the current study found that diabetes, elevated lipids, and urinary symptoms are associated with the presence of prostate cancer, whereas diabetes and altered lipids are associated with the presence of female breast cancer. Prior research demonstrated mixed findings on the associations between lipids and the risk of breast or prostate cancer. A meta‐analysis has found that serum lipids are generally inversely associated with the risk of female breast cancer [21]. Specifically, triglycerides demonstrated an overall negative association with the risk of breast cancer, and HDL cholesterol exhibited an inverse association with the risk of breast cancer only among postmenopausal females [21]. Another Mendelian study suggested the individual harmful effects of genetically predicted testosterone, HDL cholesterol, and IGF‐1 on female breast cancer risk [22]. In the current study, LDL cholesterol showed an inverse association but triglycerides exhibited a positive association. However, the association of HDL cholesterol was not significant. On the other hand, previous meta‐analyses in observational studies [23] and Mendelian randomization studies [56] did not support any association between lipids and prostate cancer risk. Nevertheless, a Finnish study with a median of 17‐year follow‐up [24] found that hypercholesterolemia appears to be associated with an increased risk of prostate cancer in the short term (3 years) but a decreased risk in the long run (20 years). Findings of the current study found that circulating lipids (LDL cholesterol, HDL cholesterol, and triglycerides) uniformly exhibit individual positive associations with the presence of prostate cancer, where HDL cholesterol demonstrates the strongest association. Furthermore, a recent meta‐analysis [32] revealed a high prevalence of prostate cancer among males with macroscopic or microscopic haematuria. This is consistent with the findings of the current study suggesting the frequent co‐occurrence of haematuria and prostate cancer in males.

There are some potential public health implications of the present study. First, findings do not support the associations between diabetes and incidence of the female breast or prostate cancer. Patients with incident diabetes do not appear to have an elevated risk of developing female breast or prostate cancer when compared to those without diabetes. Rather, the observed co‐occurrence could be due to shared risk factors or heightened surveillance for diabetes upon cancer diagnosis. Consistent with the current pilot breast cancer screening criteria in Hong Kong, the presence of diabetes alone is not considered as a high‐risk factor for screening. Second, while evidence for the link between smoking and prostate cancer is less conclusive, this study supports smoking as a potential risk factor for prostate cancer. Further studies are warranted to examine whether tobacco prevention is additionally beneficial for prostate cancer prevention. Third, patients with prostate cancer exhibit uniformly elevated levels of different lipids and urinary symptoms. Future research is warranted to characterize the clinical profile of male patients with prostate cancer.

Strengths of the present study include a relatively large sample size, inclusion of an incident diabetes cohort, and confounding control from information available in the electronic health records. However, several limitations may exist in the current study. First, information on some potential confounders such as anthropometric measurements (adiposity indicators) or hormonal‐related factors (use of oral contraceptives, hormone replacement therapy, or direct measurements of circulating hormone levels) was not available in this study. Nevertheless, diabetes itself could be a marker of obesity [5], and current evidence does not support the association between adiposity and prostate cancer [9]. Furthermore, incorporating additional confounders such as BMI [5] is likely to attenuate the observed association towards the null. Second, some information at cancer diagnosis such as hormone receptor status, cancer staging, or PSA level was also not available. Third, further studies with longer follow‐up time are warranted due to the long latency period of cancer. Fourth, the concurrent presence of diabetes among patients with female breast or prostate cancer could be partially attributed to detection bias. Fifth, while Mendelian randomization studies [37, 57] suggest a limited genetic role of type 2 diabetes on cancer risk, future research is warranted to generalize the findings to other populations.

## 5 Conclusion

This study does not find sufficient evidence for the association between diabetes and incidence of female breast or prostate cancer. Nevertheless, findings suggest smoking as a potential risk factor for prostate cancer. In addition, diabetes, elevated lipid levels, and urinary symptoms appear to be associated with the presence of prostate cancer, whereas diabetes and altered lipid profile appear to be associated with the presence of breast cancer. In our Asian population, while incident diabetes alone does not appear to be associated with an increased risk of developing female breast or prostate cancer, the observed frequent co‐occurrence could be due to shared risk factors or heightened surveillance. Future research may conduct prospective studies with a longer follow‐up time and incorporate a more comprehensive list of factors (such as lifestyle and genetic factors) for confounding control.

## Author Contributions

**Eman Yee Man Leung:** writing – review and editing. **Sarah Tsz Yui Yau:** conceptualization, methodology, formal analysis, data curation, writing – original draft. **Chi Tim Hung:** conceptualization, methodology, writing – review and editing, supervision. **Eng Kiong Yeoh:** conceptualization, methodology, writing – review and editing, supervision. **Albert Lee:** writing – review and editing.

## Funding

The authors have nothing to report.

## Ethics Statement

Ethics approval for secondary data analysis was provided by the Joint Chinese University of Hong Kong—Survey and Bahavioral Research Ethics Committee (reference number: SBRE‐22‐0386). Patient consent was waived since individuals were not identifiable in this study.

## Conflicts of Interest

The authors declare no conflicts of interest.

## Supporting information


**Table S1:** Definitions of diseases using diagnosis codes.

## Data Availability

Data is not available for sharing due to restricted access.
