# Supplementary material for: Associations of Diabetes, Smoking, and Metabolic Factors With the Risk of Breast and Prostate Cancers: A Population‐Based Retrospective Cohort Study
Source: Cancer Med. 2026 Jan 26;15(2):e71556. doi: 10.1002/cam4.71556 (PMC12835555; doi:10.1002/cam4.71556)
Supplement: Supplementary file 1 — Table S1: Definitions of diseases using diagnosis codes. [file CAM4-15-e71556-s001.docx]

Supplementary Table S1. Definitions of diseases using diagnosis codes

| **Disease** | **Diagnosis code** |
| --- | --- |
| Breast cancer | ICD-10: C50 |
| Prostate cancer | ICD-10: C61 |
| Haematuria | ICPC-2: U06  ICD-10: R31 |
| Cystitis | ICPC-2: U71  ICD-10: N30 |
| Chronic obstructive pulmonary disease | ICD-10: J44, J44.8, J44.9 |
| Pneumonia | ICD-10: J12-J18 |
| Tuberculosis | ICPC-2: A70  ICD-10: A15 |
| Ischemic heart disease | ICD-10: I20-I25 |
| Cerebrovascular disease | ICD-10: I60-I69 |
| Heart failure | ICD-10: I50 |
| Chronic hepatitis B | ICD-10: B18.0, B18.1 |
| Chronic hepatitis C | ICD-10: B18.2 |
| Liver cirrhosis | ICD-10: K70.3, K71.7, K74, K74.6 |
| Fatty liver | ICD-10: K70.0, K76.0 or radiology reports (ultrasonography or computed tomography or magnetic resonance imaging) |

ICD-10, International Classification of Disease 10^th^ revision; ICPC-2, International Classification of Primary Care 2^nd^ edition.
